# A rehabilitation intervention to improve recovery after an episode of delirium in adults over 65 years (RecoverED): study protocol for a multi-centre, single-arm feasibility study

**DOI:** 10.1186/s40814-023-01387-y

**Published:** 2023-09-15

**Authors:** Louise Allan, Abby O’Connell, Shruti Raghuraman, Alison Bingham, Abigail Laverick, Kirstie Chandler, James Connors, Benjamin Jones, Jinpil Um, Sarah Morgan-Trimmer, Rowan Harwood, Victoria A. Goodwin, Obioha C. Ukoumunne, Annie Hawton, Rob Anderson, Thomas Jackson, Alasdair M. J. MacLullich, Sarah Richardson, Daniel Davis, Lesley Collier, William David Strain, Rachael Litherland, Jon Glasby, Linda Clare

**Affiliations:** 1 University of Exeter Medical School, University of Exeter, Exeter, UK; 2 School of Health Sciences, University of Nottingham, Nottingham, UK; 3 Nottingham University Hospitals NHS Trust, Nottingham, UK; 4 Department of Ageing and Rehabilitation, University of Exeter, Exeter, UK; 5 Department of Health and Community Sciences, Faculty of Health and Life Sciences, National Institute for Health and Care Research (NIHR) Applied Research Collaboration (ARC) South West Peninsula (PenARC), University of Exeter, Exeter, EX1 2LU UK; 6 Health Economics Group, University of Exeter Medical School, Exeter, UK; 7 Exeter HS&DR Evidence Synthesis Centre, Institute of Health Research, University of Exeter Medical School, University of Exeter, Exeter, UK; 8 Institute of Inflammation and Ageing, University of Birmingham Research Laboratories, Queen Elizabeth Hospital, Mindelsohn Way, Edgbaston, Birmingham, B15 2WD UK; 9 Scottish Hip Fracture Audit (SHFA), NHS National Services Scotland, Edinburgh, UK; 10 Ageing and Health Group, Usher Institute, University of Edinburgh, Edinburgh, UK; 11 AGE Research Group, Translational and Clinical Research Institute, Newcastle University, Newcastle Upon Tyne, UK; 12 MRC Unit for Lifelong Health and Ageing at UCL, London, WC1E 7HB UK; 13 Faculty of Health and Well-Being, University of Winchester, Winchester, SO22 4NR UK; 14 Diabetes and Vascular Medicine Research Centre, Institute of Biomedical and Clinical Science, College of Medicine and Health, University of Exeter, Exeter, EX2 5AX UK; 15 Innovations in Dementia, Exeter, UK; 16 School of Social Policy, University of Birmingham, Birmingham, UK

**Keywords:** Feasibility study, Delirium, Dementia, Intervention, Process evaluation, Rehabilitation

## Abstract

**Background:**

Delirium affects over 20% of all hospitalised older adults. Delirium is associated with a number of adverse outcomes following hospital admission including cognitive decline, anxiety and depression, increased mortality and care needs. Previous research has addressed prevention of delirium in hospitals and care homes, and there are guidelines on short-term treatment of delirium during admission. However, no studies have addressed the problem of longer-term recovery after delirium and it is currently unknown whether interventions to improve recovery after delirium are effective and cost-effective. The primary objective of this feasibility study is to test a new, theory-informed rehabilitation intervention (RecoverED) in older adults delivered following a hospital admission complicated by delirium to determine whether (a) the intervention is acceptable to individuals with delirium and (b) a definitive trial and parallel economic evaluation of the intervention are feasible.

**Methods:**

The study is a multi-centre, single-arm feasibility study of a rehabilitation intervention with an embedded process evaluation. Sixty participants with delirium (aged > 65 years old) and carer pairs will be recruited from six NHS acute hospitals across the UK. All pairs will be offered the intervention, with follow-up assessments conducted at 3 months and 6 months post-discharge home. The intervention will be delivered in participants’ own homes by therapists and rehabilitation support workers for up to 10 intervention sessions over 12 weeks. The intervention will be tailored to individual needs, and the chosen intervention plan and goals will be discussed and agreed with participants and carers. Quantitative data on reach, retention, fidelity and dose will be collected and summarised using descriptive statistics. The feasibility outcomes that will be used to determine whether the study meets the criteria for progression to a definitive randomised controlled trial (RCT) include recruitment, delivery of the intervention, retention, data collection and acceptability of outcome measures. Acceptability of the intervention will be assessed using in-depth, semi-structured qualitative interviews with participants and healthcare professionals.

**Discussion:**

Findings will inform the design of a pragmatic multi-centre RCT of the effectiveness and cost-effectiveness of the RecoverED intervention for helping the longer-term recovery of people with delirium compared to usual care.

**Trial registration:**

The feasibility study was registered: ISRCTN15676570

**Supplementary Information:**

The online version contains supplementary material available at 10.1186/s40814-023-01387-y.

## Background

Delirium is an acute disorder of cognitive function, particularly common in hospitalised older people. The primary feature is disturbance in attention and awareness accompanied by impairments in cognition and changes in behaviour. People with delirium can present with restlessness, withdrawal and agitation and often experience visual hallucinations and delusions. Delirium arises as a direct physiological consequence of another medical condition and has an acute onset and fluctuating course [1].

Delirium is associated with poor outcomes: increased length of stay in hospital, hospital-acquired complications, distress, poor functional recovery, and increased mortality [2–9]. People with delirium often struggle to communicate [10, 11], leading to significant distress and negative emotions among family caregivers [10, 12, 13]. This can result in a breakdown in the caregiver-patient relationship, leaving family carers feeling helpless and overwhelmed [7]. In addition to being highly distressing to people with delirium and their caregivers, delirium is expensive with total inpatient costs attributable to delirium ranging from £12,575 to £49,689 per patient-episode [14]. Cognitive and functional deficits can persist for months after an episode of delirium and some people may never recover, with 21% having persistent delirium after 6 months [15]. Delirium is also strongly associated with subsequent dementia and a decline in cognitive function in those with and without dementia [3, 9, 16–18]. People who do not fully recover from delirium are more likely to require an increased level of care or institutionalisation [5, 19, 20]. Delirium can cause a rapid decline in functional status [11, 21], and several studies have shown that functional impairment can last up to 1 year [22–25]. Studies which have shown a decrease in performance of activities of daily living (ADL) after delirium suggest that people have ongoing care needs which may be ameliorated by rehabilitation [2, 26].

Little is known about the support needs of people with delirium and their carers or the support currently available after leaving hospital. Previous research has addressed prevention of delirium in hospitals and care homes, and there are guidelines on short-term treatment of delirium during admission [27, 28]. However, no studies have addressed the problem of longer-term recovery after delirium. Thus, an evidence-based and cost-effective intervention is needed to promote recovery after delirium.

Here we report the protocol for a study designed to test the feasibility of (i) a rehabilitation intervention in older people diagnosed with delirium during acute hospital admission and (ii) the methods for a future definitive randomised controlled trial (RCT) with parallel economic evaluation.

## Intervention development

People with cognitive impairment or dementia are often not referred for rehabilitation due to a perception that they do not have “rehabilitation potential”; however, rehabilitation interventions can be readily applied to people with cognitive impairment and can be effective [29, 30]. These findings suggest that there is a gap in evidence that limits advances in rehabilitation addressing longer-term recovery after delirium. To address this gap, we have developed a multicomponent intervention to improve recovery after a delirium episode with the aim of improving longer-term recovery after delirium for older people. The development work followed the principles of the Medical Research Council guidance on developing and evaluating complex interventions [31]. We first conducted a realist review to examine current evidence for, and theory underpinning, interventions to help people recover after delirium [32]. This led to the development of an initial programme theory, which provided a theoretical and conceptual framework to explain the core elements of a community-based delirium intervention. The programme theory also identified how elements were related to each other, how they could be implemented by practitioners and how mechanisms of impact were operated to produce outcomes. Following this, a qualitative interview study was conducted with key stakeholders including older people, carers and healthcare professionals to investigate the perceived rehabilitation needs of older people who had delirium during a hospital stay. The findings were presented to an expert panel of key stakeholders consisting of healthcare professionals, older people with lived experience of delirium and caregivers. Their insights on the findings informed the refinement of the programme theory. Throughout the iterative development process, we actively engaged and sought insights from older people with lived experience of delirium and caregivers, ensuring their perspectives were incorporated into the intervention design.

The refined programme theory is underpinned by six core intervention elements and is represented in summary form in a logic model (see Additional file 1: RecoverED logic model) The elements are (1) providing guidance around good medical care including nutrition and hydration, sleep hygiene and optimal physical environment to enable recovery; (2) regularly monitoring active treatment through coordinating with relevant services, such as the general practitioner (GP), to address any physiological or medical symptoms or conditions; (3) focused psychoeducation in the form of information about delirium and its presentation; (4) supporting recovery from any emotional or mental distress through talking with skilled helpers to normalise and legitimise responses to symptoms, active therapeutic listening and motivating and encouraging social contact and interaction; (5) cognitive rehabilitation to enhance functional ability through guided practice of everyday activities with application of enhanced learning and compensatory strategies; and (6) physical rehabilitation using planned, structured and repetitive strength and balance activities, and practice of functional activity to improve physical health. These elements, in addition to the core elements, should be delivered in a style which is expected to be important to the success of the intervention. This should include (1) a person-centred approach that acknowledges the unique identities of each participant and treats them with dignity, compassion and respect; (2) tailoring the intervention to individual needs, preferences and abilities; (3) engaging carers throughout the intervention; (4) integrating intervention goals and activities into daily life to improve engagement; (5) ensuring quality and continuity of relationships of care; and (6) integrating with local services to broaden service delivery and fill service gaps. The programme theory will be tested, developed and refined during the feasibility study and will inform the intervention design and data collection plans for a definitive RCT.

## Objectives

The specific objectives of the study are as follows:

### Primary objectives

1. Assess the feasibility of the rehabilitation intervention in older people who have had delirium to determine whether the intervention is acceptable to them and their carers.

### Secondary objectives

1. Examine the acceptability of the intervention for individuals with diverse characteristics via a process evaluation.
2. Test the feasibility of processes for collecting data required to inform the choice of primary and secondary outcome measures for the definitive RCT.
3. Test the ability to collect the data required to undertake an economic evaluation alongside a future definitive RCT.
4. Engage in iterative refinement of the intervention for the definitive RCT.

## Methods/design

### Study design

This study is a multi-centre, single-arm feasibility study of a rehabilitation intervention with an embedded process evaluation, the results of which will be reported in line with the CONsolidated Standards of Reporting Trials (CONSORT) extension [33], the Standard Protocol Items: Recommendations for Interventional Trials (SPIRIT) statement (see Additional file 2) [34], and the Template for Intervention Description and Replication (TIDieR) checklist (see Additional file 3) [35] to ensure clear and transparent reporting of the study methods and results. Participants with delirium and carers will be recruited in pairs. All participants will be offered the intervention and assessed at baseline while participants with delirium are still in-patients in the acute hospital and follow-up assessments will be conducted at 3 months and 6 months post-discharge home. Feasibility and acceptability of the intervention and outcome measures will be assessed using a mixed methods approach.

### Study setting and participants

Sixty participants with delirium and their carers will be recruited from six acute hospitals in the UK that provide care for older people with delirium. The intervention will take place in participants’ own homes. Follow-up will take place in the participants’ homes or at the hospital or NHS clinic, depending on participant preference and abilities, and the capacity of the local research delivery team.

### Participant eligibility

Eligible participants will be people who have been admitted to a participating acute hospital and have a clinical diagnosis of delirium with symptoms persisting for at least 48 h. Individuals with delirium either with or without dementia will be screened for suitability to take part in the study. Carers will be screened and recruited to the study as a pair with the participant with delirium. Participants with communication difficulties due to advanced dementia or aphasia, or who do not have an appropriate carer, will not be included. The reasons for exclusion are that people with advanced dementia or aphasia are unlikely to benefit from the intervention and that the carer is required to complete outcome measures.

#### Inclusion criteria for participants with delirium

1. Aged over 65 years.
2. Admitted to an acute hospital.
3. Clinical diagnosis of delirium with symptoms lasting for more than 48 h.
4. Expected to be living in a private dwelling after discharge from hospital.
5. Has a carer who is willing to assist with completion of outcome measures.
6. Has the capacity to provide informed consent to participate, or in accordance with current legislation, has a consultee who can give an opinion on participation (in England), or has a legal representative who is able to give informed consent on behalf of the participant (in Scotland).

#### Exclusion criteria for participants with delirium

1. Diagnosis of delirium cannot be confirmed during the hospital admission.
2. Unable to communicate verbally due to advanced dementia or aphasia.
3. Carer declines to participate
4. Undergoing end of life care.
5. Participating in another intervention study.

#### Inclusion criteria for carer participants

1. Family member or friend of the person with delirium who is going to take part in the study.
2. In contact with the person with delirium for at least 1 h per week.
3. Able to communicate in English sufficiently well to complete the proxy outcome measures.
4. Has capacity to provide informed consent and consents to participate.

There are no exclusion criteria for carers.

### Recruitment

Potential participants will be in-patients, primarily on older people’s medical wards, general medical wards and trauma and orthopaedics wards. To optimise resource utilisation for screening, we have opted to focus on wards with the highest prevalence and incidence of delirium. Participants will have already been screened for delirium as part of standard care using a suitable well-established method such as the Confusion Assessment Method or the 4AT Rapid Clinical Test for Delirium Detection [36]. Eligibility will initially be assessed through discussion with the clinical care team to screen out participants who are clearly ineligible (≤ 65 years of age, care home residents, receiving palliative care). Potentially eligible participants will be assessed by the clinical care team to confirm a diagnosis of delirium. The detailed assessment for delirium is conducted as part of good practice as recommended in the UK by the National Institute for Health and Care Excellence (NICE) for people who screen positive for delirium [28]. Participants who have not been delirious for 48 h at the time of initial delirium screening will be reassessed after 48 h post-diagnosis to confirm persistent delirium. Where a participant is eligible to participate subject to carer agreement, carers will be screened to confirm they meet inclusion criteria. This will be established by discussion with the participant’s clinical care team.

### Consent process

Participants will be required to give informed consent at the time of recruitment. If due to health conditions the participant is unable to provide informed consent, an appropriate personal consultee/legal representative will be approached to provide consultee advice (England) or agreement (Scotland). The study exclusively uses personal consultees, and these individuals are provided with separate information sheets outlining their specific role.

All eligible participants will be informed that participation in the study is voluntary and that they may withdraw with no prejudice to their care if they choose not to participate or chose to withdraw from the study after giving consent. The participant or consultee/legal representative will be given the opportunity to read the participant/consultee/legal representative information sheets and ask any questions before participating in the study. The participant will be given a copy of the signed consent form to keep, and a copy will be filed in the investigator site file. If during the intervention period a participant who previously lacked capacity regains capacity to give informed consent, written informed consent will be obtained from the participant to affirm willingness to continue in the study, or the participant will be withdrawn from the study upon request. Additional sections in the main consent form will cover agreement from participants with delirium and carers to participation in the embedded process evaluation. Participation in the process evaluation is optional and declining participation will not affect a participant’s ability to take part in the main intervention study.

### Baseline assessments

Baseline assessments will be undertaken by a researcher (research nurse or equivalent) who is appropriately trained and authorised to work on the study. Baseline assessments will be conducted after the participants have consented to take part in the study and while the participant with delirium is still an in-patient in the acute hospital. Assessments and participant-reported outcome measures will be completed as shown in Table 1 and in Additional file 4. The researcher will support the participants to complete paper questionnaires. Medical history (taken from medical records), demographic information (e.g. age, sex, ethnicity), and contact details will be collected prior to discharge from hospital.

**Table 1:**
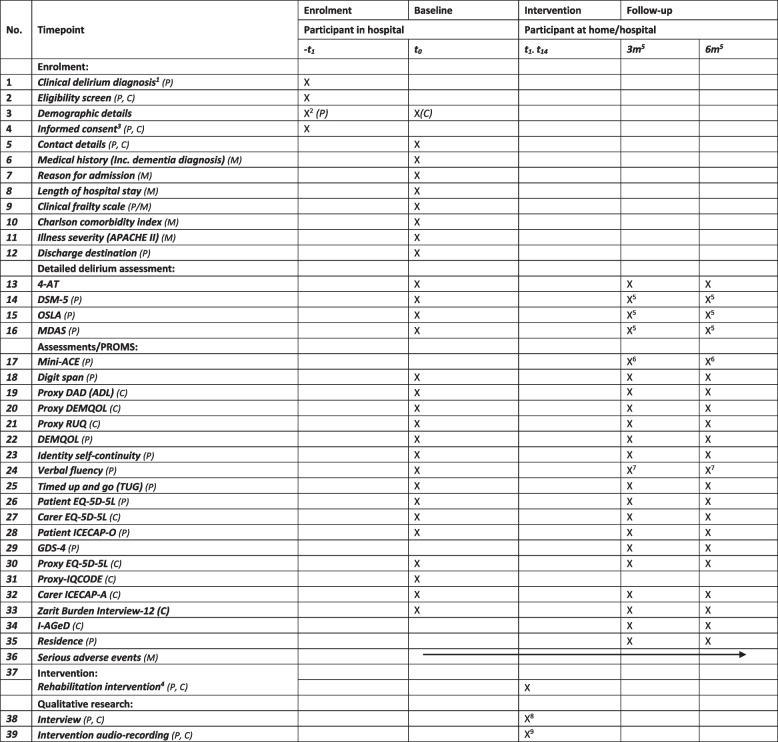
Schedule of enrolment, intervention, assessments and data collection

*P* participant with delirium, *C* carer participant, *M* medical records, *3m* 3-month follow-up, *6m* 6-month follow-up, *t(week)* time point in weeks

^1^Standard care

^2^Anonymised data prior to consent

^3^Informed consent or proxy equivalent as appropriate to England or Scotland regulations

^4^Includes initial home assessment within 2 weeks of discharge home. Delivered in participant’s private home with a support worker, dose up to 10 sessions across 12 weeks after the initial home assessment

^5^Completed only if evidence of persistent delirium if 4AT score ≥ 4 at visit

^6^Mini-ACE completed only if 4AT score < 4 at follow-up visit

^7^Completed as standalone item if mini-ACE not completed at visit (see footnote 6 above)

^8^One-off interview to be conducted face-to-face-, online or by telephone with a sample of 15–20 participants/patient-carer pairs and 20–24 healthcare professionals shortly after the end of the intervention

^9^A sample of 15–20 intervention sessions will be audio-recorded to assess fidelity of the delivery of the intervention

### Follow-up

All participants will be followed up 3 months and 6 months post-discharge to conduct the assessments shown in Table 1. The permissible time window for follow-up visits is ± 2 weeks for both 3- and 6-month follow-ups. This specific time frame was chosen to strike a balance between ensuring sufficient time between data collection at different waves and allowing some flexibility for data collection. By having a ± 2-week time frame, it ensures that the follow-up visits are appropriately spaced and preventing data overlap.

The researcher and participants will complete pseudonymised paper Case Report Forms (CRFs) and questionnaires. All collected data will be entered into the electronic data capture (EDC) system via a Research Electronic Data Capture (REDCap) electronic platform by the researcher.

### Intervention

The RecoverED intervention is based on rehabilitation activities designed to support recovery after delirium at home. The intervention will be initiated with a home assessment visit from a community physiotherapist (PT) or occupational therapist (OT). The home assessment visit will take place within 2 weeks of discharge from hospital. It will take up to 90 min and include the following:

- Reviewing the care package the participant is already receiving
- Assessing the safety of the home environment
- A functional and mobility assessment
- Providing advice about how to access relevant resources in the community to meet an identified unmet need
- Reviewing medication and referring to the participant’s GP if specific medications of concern are identified (therapists will be provided with a list of medications to indicate when GP referral is required)
- Discussion regarding goals and a brief summary of the intervention

A proforma report will be completed by the PT/OT at the home assessment visit to record the participant’s current abilities, impairments and personal goals so the intervention can be tailored to individual needs. The interventions will be agreed with the participant with delirium and carer and reviewed with a rehabilitation support worker (RSW) to plan the session details. The RSW will deliver up to 10 home intervention sessions over 12 weeks following the home assessment visit. One of the intervention sessions will serve as a review session where the OT or PT will attend along with the RSW to review the participant’s goals and recovery progress.

Participants will be provided with a paper-based recovery record to keep for the duration of the study. The recovery record will be used by the RSW and the participants to plan and record activities and keep a timeline of their progress.

### Intervention training and support

The intervention training programme was developed through a collaborative effort of the core study team, including OT, PT and CP. The team held biweekly meetings to ensure a systematic and evidence-based approach to the training programme’s development. Guided by insights from the programme theory, the training content was designed to equip the intervention delivery team (OTs, PTs and RSWs) with the necessary knowledge and skills for effective intervention delivery. The comprehensive training package covers various modules (see Additional file 5), and these modules will be reviewed and refined based on feedback gained from the process evaluation and through Participatory Action Research (PAR) informed focus groups held 12 weekly.

Training sessions will be recorded and made available to the intervention delivery team at the site. The initial intervention training package will take approximately 8 h. Intervention delivery review sessions will be offered to the intervention delivery teams by the core research team (OT, PT and qualitative researcher) every 3 weeks. The intervention review sessions will take approximately eight cumulative hours per site over the course of the intervention delivery period.

### Feasibility outcomes

The following feasibility outcomes will be assessed:

1. The number of people with delirium identified on hospital wards
2. The proportion (and number) of people with delirium who meet the eligibility criteria
3. The proportion of eligible people with delirium who agree to participate in the study*
4. The proportion of carers who agree to participate in the study
5. The proportion of participating people with delirium who start the intervention
6. The proportion of participating people who complete ≥ 60% of the intervention sessions*
7. The proportion of participating people with delirium who remain in the study until final follow-up at 6 months*
8. The proportion of people with delirium providing valid outcome data for each primary and secondary outcome measures at 3- and 6-month follow-ups
9. The acceptability of the intervention assessed during the process evaluation*
10. The standard deviation and 6-month follow-up rate for the proposed primary outcome, in order to either verify or inform revision of the proposed sample size calculation for the definitive RCT

*Feasibility outcomes marked with an asterisk will be used to determine whether the study meets the criteria for the definitive RCT (see Table 2 below).

**Table 2:** Progression criteria for RCT

Domain	Proceed with RCT (green light)	Do not proceed (red light)
Recruitment	≥ 25% of eligible participants consenting (or consultee agreeing) to feasibility study	< 10% of eligible participants consenting to feasibility study
Completion of intervention	≥ 70% participants attend ≥ 60% of sessions as planned	< 30% participants attend ≥ 60% of sessions as planned
Retention	Retention of ≥ 60% of recruited participants for key outcome data at 6 months	Retention of < 50% of consented participants for provision of key outcome data at 6 months
Intervention acceptability	Evidence from the process evaluation that the intervention can be delivered with fidelity and that it is acceptable to individuals with diverse characteristics	Evidence from the process evaluation that the intervention cannot be delivered with fidelity and that it is not acceptable to individuals with diverse characteristics
Intermediate targets will be defined as amber and will be reviewed by the Programme Steering Committee and funder		

These feasibility outcomes will also be considered separately for those with and without dementia. Additional file 6 shows a list of objectives matched with feasibility outcomes.

### Economic evaluation

As part of the feasibility study, methods will be trialled for capturing:

i) The resources required to deliver the intervention. This will be assessed via participant-level CRFs and discussion with the intervention developers and providers. This is expected to include staff time (e.g., OT, PT, RSW), travel, training, supervision and materials.
ii) Health, social and wider care service resource use. Data will be collected via a proxy Resource Use Questionnaire (RUQ). Items from the CORE Items for a Standardized Resource Use Measure (ISRUM) will be included in the RUQ, which will also draw on measures in the Database of Instruments for Resource Use Measurement [37, 38].
iii) Health economic outcomes relating to health-related quality of life and wellbeing. Summaries of EQ-5D-5L [39] and EQ-5D-5L Proxy Version 2 (carers), DEMQOL and DEMQOL-proxy (carers), ICECAP-O, and ICECAP-A (carers) data will be reported.

### Embedded process evaluation

An embedded process evaluation will be conducted to examine implementation, mechanisms of impact and context of the intervention. It will be structured using the logic model, based on the programme theory developed from the initial realist review and realist qualitative study.

Healthcare support workers delivering the intervention will complete a CRF at each intervention session to document the activities undertaken by the participant, any issues with completing the session and the time spent undertaking the intervention. In addition, a purposive sample of 15–20 intervention sessions will be audio-recorded by the RSW using an encrypted audio-recorder. The audio-recordings will be used to assess the intervention delivery against a fidelity checklist to ensure treatment fidelity and assess the intervention approach. This will ensure that the intervention is tailored and personalised to individual needs, preferences, abilities and that it actively involves the participation of the carer.

Participants and healthcare professionals will be invited to take part in qualitative interviews. Qualitative data will be collected by the study qualitative researcher who will be a university employee and not involved in the delivery of the intervention:

- In-depth, semi-structured qualitative interviews will be conducted with 15–20 participant/participant-carer pairs, sampled purposively to include individuals with diverse characteristics. Participants will be interviewed once for up to 60 min via telephone or online shortly after the intervention.
- In-depth, semi-structured interviews will be conducted with 20–24 professionals in three participating sites, by telephone or online. Professionals will be sampled to include the range of roles involved in planning and delivery of services in order to inform the potential scalability of the intervention.

Interviews will explore fidelity, acceptability, intervention recruitment and retention, mechanisms of impact, views about the optimal dose of the intervention, and contextual factors affecting the intervention. This information will refine the intervention theory developed previously and inform the design for the definitive trial. Qualitative data will be analysed using realist methods with NVivo The analysis will be conducted in several stages, first deductively coding whole or partial CMOCs (context, mechanism, outcome configurations) from previous programme theory and also using inductive coding to refine CMOCs. CMOCs will then be grouped and refined and used to refine the programme logic model. Findings will be compared between participants with and without a dementia diagnosis. Quantitative data on reach, retention, fidelity and dose will also be collected and summarised using descriptive statistics in Excel, SPSS or a database programme such as Access. The process evaluation findings will then be integrated using a triangulation strategy across the various datasets by two members of the qualitative research team. We will use one or more triangulation tables to display cases (individuals, sites) in rows and categories (e.g. fidelity, acceptability) in columns. Categories will be developed based on key areas of interest for the process evaluation but will also be constructed inductively drawing on emerging study findings. Cells in the table will be populated with both qualitative and quantitative findings. The tables will then be explored to identify patterns within cases (rows) and categories (columns) and to also examine the relationships between different cases and categories. Patient and Public Involvement and Engagement (PPIE) panel members will be invited to provide comments and feedback.

In addition, we will undertake regular iterative revision of the intervention during the intervention period using a Participatory Action Research approach [40]. Therapists and RSWs will be asked to join an online focus group every 6 weeks to reflect on their experience of the intervention so far. Therapists will discuss the feasibility and acceptability of the intervention and their ideas for developing it, including any factors which might affect fidelity in the main trial. Emerging findings from participant interviews will also be discussed in the focus groups, as well as ongoing quantitative findings on reach, dose and retention. The focus groups will be facilitated by our research team therapists and qualitative researcher and recorded for qualitative analysis. At the end of the intervention period, all findings will be synthesised to achieve the final intervention for testing in the definitive RCT.

### Progression criteria

Table 2 illustrates the feasibility outcomes that will be used to determine whether the study meets the criteria for progressing to a definitive RCT. The targets were pragmatically established in consultation with the funder and Programme Steering Committee to ensure they align with the potential success of a subsequent RCT.

### Planned outcomes for a definitive RCT

In this single-arm feasibility study, we will collect data on the outcome measures proposed for use in a future definitive RCT (Table 3). This will enable us to test whether the data can be collected, but data will be presented descriptively only. It is possible that some outcome measures will be changed as part of revision of the study prior to undertaking the definitive RCT.

**Table 3:** Planned future RCT outcomes

Measure	Description	Time point
**Proposed primary**		
Activities of daily living (ADL) with the Disability Assessment for Dementia (DAD) [41]	This will be assessed by the DAD which has a 40-item scale and address a range of functional domains. 17 items address basic ADLs and 23 items relate to Instrumental ADLs. Scores range from 0–100% witch higher percentage scores representing greater competence in ADLs	Baseline, 6 months
**Secondary**		
Activities of daily living (ADL)	As described above for primary outcome	Baseline, 3 months
Mobility	This will be assessed using the Timed Up and Go (TUG). Participants stand up and walk 3 m, turn around and walk back. The time taken to complete it is recorded. The test is scored as time in seconds	Baseline, 3 months, 6 months
Delirium persistence or recurrence	This will be assessed by DSM5 criteria [42], with some additional enhancements including use of the Informant Assessment of Geriatric Delirium scale (I-AGeD) [43]	Baseline, 3 months, 6 months
Attention	Assessment of attention using months of the year backwards. Participants will be asked to recite the months of the year backwards from December to June, and a score ranging from 0–7 represents number of months successfully recited before failure. A score of 7 indicates correct recital	Baseline, 3 months, 6 months
Level of arousal	Observational Scale of level of Arousal (OSLA) [44] will be used to assess the level of arousal in people with delirium	Baseline, 3 months, 6 months
Cognition	This will be assessed with mini ACE (Mini-ACE) [45] which consists of 5 items and has a maximum score of 30	Baseline, 3 months, 6 months
Verbal fluency	Verbal fluency will be assessed using the ‘Animals’ item from the mini-ACE. The score represents the correct number of animals stated in 1 min	Baseline, 3 months, 6 months
Self-continuity	Single item question to assess sense of self-continuity. Scored on a Likert scale (Strongly disagree/disagree/neither agree nor disagree/agree/strongly agree)	Baseline, 3 months, 6 months
Verbal short-term and working memory	This will be assessed with the Digit span test (Forward Digit Span and Reverse Digit Span). Participants repeat a sequence of numbers in forward or reverse order. The length of digit span repeated correctly will be scored 0–8	Baseline, 3 months, 6 months
Mood assessment	This will be assessed using Geriatric Depression Scale-4 (GDS-4) [46]. The 4 binary item scale will be used which has been validated in older people and people with dementia. The items will be summed to form a total score with a potential range of 0–4	3 months, 6 months
Wellbeing	This will be assessed using the ICEpop CAPability measure for Older people (ICECAP-O) [47]	Baseline, 3 months, 6 months
Residence category	Residence types (own home, living with family, assisted living/warden supported, care home without nursing, care home with nursing or other residence type)	3 months, 6 months
Patient health-related quality of life (HRQL)	This will be assessed using the EQ-5D-5L and EQ-5D-5L proxy [39]. Participant responses to the EQ-5D-5L will be converted to health state values (HSVs) to provide quality-adjusted life-years (QALYs)	Baseline, 3 months, 6 months
Patient HRQL	This will be assessed with the DEMQOL and DEMQOL-Proxy [48]	Baseline, 3 months, 6 months
Carer burden	This will be assessed using the Zarit Burden Interview 12 (ZBI-12) [49]. 12 Items (rated 0–4) are summed to give a total score in the range 0–48	Baseline, 3 months, 6 months
Carer quality of life	This will be assessed using the EQ-5D-5L. Carers participating in this study will be asked to report their own HRQOL, using the EQ-5D-5L, as described above	Baseline, 3 months, 6 months
Carer wellbeing	This will be assessed using the ICEpop CAPability measure for Adults (ICECAP-A) [50]	Baseline, 3 months, 6 months
Resource use	Data will be collected via a proxy Resource Use Questionnaire (RUQ). The RUQ will be informed by the ‘CORE Items for a Standardised Resource Use Measure (ISRUM)’ [37] and the Database of Instruments for Resource Use Measurement [38], and will be designed in collaboration with the PPIE group	Baseline, 3 months, 6 months

### Adverse events management

A risk assessment has identified that this is a low-risk intervention study. Non-serious adverse events (AEs) will not be recorded. We will record serious adverse events (SAE) only for the participants with delirium. All deaths (from any cause) and hospitalisations due to falls, fractures or musculoskeletal injury will be recorded as SAEs. Other SAEs will not be recorded. This rationale is based on several factors, including the expected frequency of other SAEs in the frail population under study and the potential burden that recording these events may impose without providing significant informative insights relevant to the study objectives.

SAEs will be reported to the study sponsor. Events which are unexpected and causally related to the intervention—termed related unexpected SAEs (RUSAE)—will be reported to the research ethics committee and the programme steering committee within 7 days of the sponsor being made aware of the event. Research staff at sites will review the participants’ medical records before each 3-month and 6-month follow-up visit and record all events that are reportable SAEs in the study EDC system. Research staff conducting follow-up visits will also ask participants whether any reportable SAEs have occurred.

### Sample size calculation

As a single-arm feasibility study, the aim is not to determine the effectiveness of the intervention, but rather to estimate key feasibility parameters to inform the design of a subsequent definitive randomised trial. As a result, no formal power calculation has been undertaken to determine the required sample size. Instead, the aim is to recruit a total of sixty participants to the study. If the value of key feasibility parameters, such as the follow-up rate and the percentage of participants attending at least 60% of the intervention sessions, is 70%, sixty participants will allow estimation of the parameter with a 95% confidence interval of 57 to 81% [51].

### Statistical analysis

Descriptive statistics will be used to summarise demographic and outcome data. Continuous data will be reported as means and standard deviations, or as medians and interquartile ranges if the data are skewed. Categorical data will be reported using numbers and percentages. A CONSORT flow diagram will be produced to illustrate the flow of participants through the study. The number of participants approached, eligible, consented and recruited, and assessed at baseline, 3 months and 6 months will be reported, along with the number of participants withdrawn or lost to follow-up between each data collection time point. The reasons for ineligibility, eligible participants not being recruited and the reasons for withdrawal where available will also be reported. The feasibility outcomes (e.g., percentage followed up at 6 months) will be reported with 95% confidence intervals overall and separately for participants with and without dementia. A comprehensive statistical analysis plan will be developed, refined and reviewed by both the trial management group and the programme steering committee before receiving final approval from the statistician and chief investigator, prior to commencing the analysis.

### Data management

Data will be collected onto paper CRFs and entered into a secure online study EDC system, with the exception of SAEs data which will be recorded directly into the EDC system. Data entered into the EDC system will be accessed by authorised personnel only. Audio-recordings of interviews and intervention sessions will be transcribed, transferred using a secure file transfer system and stored on restricted-access folders on secure University of Exeter servers accessed only by authorised members of the study team. Study documents will be archived for 5 years after the end of the study. After 5 years, all personally identifiable data will be securely destroyed upon authorisation from the Sponsor. The anonymised quantitative dataset will be stored indefinitely for the purposes of future ethically approved research.

### Data protection and confidentiality

We will adhere to the Data Protection Act 2018 when collecting, storing and reporting data. Study data will be reported anonymously so that it will not be possible to identify any individual taking part in the study. Each participant will be assigned a unique ID number. Personally identifiable data will be collected and stored separately to research data and will only be accessible to authorised members of the research team.

### Study management

The Royal Devon University Healthcare NHS Foundation Trust is the sponsor for this study. The study will be supported by the Exeter Clinical Trials Unit (ExeCTU), a UKCRC registered Clinical Trials Unit. ExeCTU will lead a combination of remote and central monitoring.

### Study governance

#### Programme Steering Committee

A Programme Steering Committee (PSC) will provide independent oversight for the study, ensuring it is conducted according to the standard set out by the UK Policy Framework for Health and Social Care Research. A separate data monitoring committee will not be convened for this study. The PSC will fulfil the role of a study steering committee and data monitoring committee and will review data completeness, data quality and accumulating safety data at agreed intervals throughout the study.

### Dissemination policy

We aim to publish the results in an open access journal within 24 months of study completion, in line with the National Institute for Health and Care Research (NIHR) guidelines. Outcome papers will adhere to CONSORT guidelines. We will work with the PPIE group to provide a lay-accessible summary of the results to all study participants. The final study report will follow the International Committee of Medical Journal Editors (ICMJE) authorship criteria.

### Patient and public involvement and engagement (PPIE)

The PPIE group have given input on the participant facing materials, intervention design and health resource use questionnaire and the study logo. One PPIE representative has been recruited to the study management group and an independent lay representative sits on the PSC. The PPIE group will meet regularly throughout the study to advise the research team of study conduct and at the end of the study they will be involved in interpreting and disseminating the results. The PPIE group is supported by Innovations in Dementia Community Interest Company.

## Discussion

The RecoverED intervention is a rehabilitation intervention for older people with and without dementia experiencing delirium and is designed to support recovery after an admission with delirium after discharge home from the acute hospital. This study is designed to test and develop the programme theory of the intervention for participants and to establish the feasibility of conducting a definitive multi-centre RCT of the effectiveness and cost-effectiveness of the intervention compared to usual care. If the trial is shown to be feasible, the study team will proceed with a definitive multi-centre RCT.

One of the main strengths of this feasibility study is that the intervention can be developed iteratively over the course of the study programme, supported by information from the embedded process evaluation, allowing the optimal approach to be adopted in future definitive RCT. Another strength is the involvement of a multidisciplinary team, advisory groups and PPIE members throughout the design process of the study. In addition, the innovative approach of including carers in the intervention and delivering it as a package to both participants with delirium and their carers is a relatively novel approach, with great potential benefit. If we can improve recovery after delirium, it may be possible both to prevent the cognitive and functional decline widely described in previous delirium research and reduce incidence of new onset dementia after delirium. This feasibility study will address this lack of evidence and inform the design of a future definitive RCT to evaluate clinical efficacy and cost-effectiveness of the RecoverED intervention.

### Study status

The feasibility study is scheduled to open for recruitment in April 2023.

### Supplementary Information

**Additional file 1.** RecoverED Logic Model.

**Additional file 2.** The Standard Protocol Items Recommendations for Trials (SPRIT) checklist.

**Additional file 3.** The TIDieR (Template for Intervention Description and Replication) Checklist.

**Additional file 4.** RecoverED study schema.

**Additional file 5.** The details of training modules.

**Additional file 6.** Feasibility objectives matched to outcomes.

**Additional file 7.** Observation protocol to measure fidelity to approach.

## Data Availability

Not applicable.

## Abbreviations

ADL: Activities of daily living
AE: Adverse event
APACHE II: Acute Physiology and Chronic Health Evaluation II
BAME: Black, Asian and Minority Ethnic
CEA: Cost-effectiveness analysis
CRF: Case-report form
DAD: Disability Assessment for Dementia
DECIDE: Delirium and Cognitive Impact in Dementia Study
DEMQOL: Dementia quality of life
DMP: Data management plan
DSM-5: Diagnostic and Statistical Manual of Mental Disorders 5th edition
EDC: Electronic data capture
ExeCTU: Exeter clinical trials unit
GDS-4: Geriatric Depression Scale 4 item
GP: General practitioner
HRA: Health Research Authority
HRQL: Health-related quality of life
HSV: Health state values
HTTPS: Hypertext transfer protocol secure
I-AGeD: Informant Assessment of Geriatric Delirium
ICECAP-A: ICEpop capability measure for adults
ICECAP-O: ICEpop capability measure for older people
ICER: Incremental cost-effectiveness ratio
ICMJE: International Committee of Medical Journal Editors
IQCODE: Informant questionnaire on cognitive decline in the elderly
ISRCTN: International Standard Randomised Controlled Trials Number
ISRUM: Items for a standardised resource use measure
MDAS: Memorial Delirium Assessment Scale
Mini-ACE: Mini-Addenbrooke’s cognitive examination
MRC: Medical research council
NICE: National Institute for Health and Care Excellence
NIHR: National Institute for Health and Care Research
NHS: National Health Service
OSLA: Observational scale of level of arousal
OT: Occupational therapist
PI: Principal investigator
PPIE: Patient and public involvement and engagement
PSC: Programme Steering Committee
PT: Physiotherapist
QALY: Quality-adjusted life years
RCT: Randomised controlled trial
REC: Research ethics committee
RecoverED: Development and testing of an intervention to improve recovery after an episode of delirium
RSW: Rehabilitation support worker
RUQ: Resource use questionnaire
RUSAE: Related unexpected serious adverse event
SAE: Serious adverse event
SMG: Study management group
SPIRIT: Standard Protocol Items: Recommendations for Interventional Trials
TUG: Timed up and go
USM: Urgent safety measure
ZBI-12: Zarit burden interview-12 item
