# Supplementary material for: A rehabilitation intervention to improve recovery after an episode of delirium in adults over 65 years (RecoverED): study protocol for a multi-centre, single-arm feasibility study
Source: Pilot Feasibility Stud. 2023 Sep 15;9:162. doi: 10.1186/s40814-023-01387-y (PMC10503099; doi:10.1186/s40814-023-01387-y)
Supplement: Supplementary file 1 — Additional file 1. RecoverED Logic Model. [file 40814_2023_1387_MOESM1_ESM.docx]

Additional file 1 RecoverED Logic Model
