# Supplementary material for: A rehabilitation intervention to improve recovery after an episode of delirium in adults over 65 years (RecoverED): study protocol for a multi-centre, single-arm feasibility study
Source: Pilot Feasibility Stud. 2023 Sep 15;9:162. doi: 10.1186/s40814-023-01387-y (PMC10503099; doi:10.1186/s40814-023-01387-y)
Supplement: Supplementary file 5 — Additional file 5. The details of training modules. [file 40814_2023_1387_MOESM5_ESM.docx]

Additional file 5 The details of training modules

| **Name of the training module**  **Length of module**  **To be completed by which members of the delivery team** | **Summary of training module content** |
| --- | --- |
| What is RecoverED?  10 Minutes  To be completed by the PT/OT & RSW | Information on the aims and processes of the study. Details regards the recovery domains and how they will be addressed through the intervention. Further details on the aspects of the RecoverED approach thought to improve recovery from delirium. |
| Delirium  10 Minutes  To be completed by the PT/OT & RSW | Definition of delirium including common signs and symptoms and the difference between dementia and delirium. Information on the current understanding of recovery from delirium. |
| Approaches and Facilitators  10 Minutes  To be completed by the PT/OT & RSW | Details on the RecoverED approach and the predicted recovery facilitators. |
| Initial Assessment  20 Minutes  To be completed by the PT & OT | How to complete the initial home assessment. |
| Planning the intervention  15 Minutes  To be completed by the PT/OT & RSW | Guidance on how to plan a personalised and person-centred intervention using the information from the initial assessment and the principles of the RecoverED approach. |
| Recovery Record  20 Minutes  To be completed by the PT/OT & RSW | Summary of the Recovery Record document and guidance on how it should be used throughout the intervention sessions. |
| Psychosocial recovery  50 Minutes  To be completed by the PT/OT & RSW | Education on how mental and emotional wellbeing can be impacted by delirium. How psychosocial recovery is being supported through the intervention. How to choose interventions based on the outcome of the initial assessment and how to deliver these in practice. |
| Cognitive recovery  50 Minutes  To be completed by the OT & RSW | Education on how cognition can be impacted by delirium, the RecoverED approach to cognitive recovery and how to design a person-cantered and goal focused intervention using the approach. |
| Physical recovery  35 Minutes  To be completed by the PT & RSW | Education on how delirium impacts physical activity. Guidance on how to select, teach and incorporate recovery activities into the intervention and into the participant’s daily life. |
| Completing the CRF and training log  5 Minutes  To be completed by the PT/OT & RSW | How to complete research paperwork and information on the importance of data quality. |
| Supervision, halfway review and final session  10 Minutes  To be completed by the PT/OT & RSW | Guidance on seeking and providing supervision, how the participant’s goals should be reviewed and adjusted according to progress and how the delivery team should conclude the final intervention session. |
