# Supplementary material for: A rehabilitation intervention to improve recovery after an episode of delirium in adults over 65 years (RecoverED): study protocol for a multi-centre, single-arm feasibility study
Source: Pilot Feasibility Stud. 2023 Sep 15;9:162. doi: 10.1186/s40814-023-01387-y (PMC10503099; doi:10.1186/s40814-023-01387-y)
Supplement: Supplementary file 6 — Additional file 6. Feasibility objectives matched to outcomes. [file 40814_2023_1387_MOESM6_ESM.docx]

Additional file 6 Feasibility objectives matched to outcomes

| **Feasibility objectives** | **Outcome Measures** | **Timepoint(s) of evaluation of this outcome measure** |
| --- | --- | --- |
| **Primary Objective** | | |
| **Objective 1:** the primary objective is to assess the feasibility of the rehabilitation intervention in older people who have had delirium to determine whether the intervention is acceptable to them and their carers | The proportion of eligible people with delirium who agree to participate in the study  The proportion of carers who agree to participate in the study  The acceptability of the intervention assessed during the process evaluation  Estimation of parameters to inform the sample size calculation for the definitive RCT | Recruitment  Recruitment  Post-intervention  Throughout |
|  |  |  |
| **Secondary Objectives** | | |
| **Objective 1:** to examine the acceptability of the intervention for individuals with diverse characteristics via a process evaluation | The acceptability of the intervention assessed during the process evaluation | Post-intervention |
| **Objective 2:** to test the feasibility of processes for collecting data required to inform the primary and secondary outcomes measures for the definitive randomised controlled trial (RCT) | The number of people with delirium identified on hospital wards  The proportion (and number) of people with delirium who meet the eligibility criteria  The proportion of participating people with delirium who start the intervention  The proportion of participating people who complete ≥60% of the intervention sessions  The proportion of participating people with delirium who remain in the study until final follow-up at 6 months*  The proportion of people with delirium providing valid outcome data for each primary and secondary outcome measure (described below) at 3 and 6 month follow ups  The estimated standard deviation and six month follow-up rate for the proposed primary outcome, in order to either verify or inform revision of the proposed sample size calculation for the definitive RCT | Recruitment  Recruitment  3 months  3 months  6 months  3 months and 6 months  6 months |
| **Objective 3:** to test the ability to collect the data required to undertake an economic evaluation alongside a future definitive RCT | The proportion of people with delirium providing valid outcome data for each primary and secondary outcome measure (as stated in Table 3) at 3 and 6 month follow ups | 3 months and 6 months |
| **Objective 4:** to engage in iterative refinement of the intervention for the definitive RCT | The acceptability of the intervention assessed during the process evaluation | Post-intervention |
