# Supplementary material for: A rehabilitation intervention to improve recovery after an episode of delirium in adults over 65 years (RecoverED): study protocol for a multi-centre, single-arm feasibility study
Source: Pilot Feasibility Stud. 2023 Sep 15;9:162. doi: 10.1186/s40814-023-01387-y (PMC10503099; doi:10.1186/s40814-023-01387-y)
Supplement: Supplementary file 7 — Additional file 7. Observation protocol to measure fidelity to approach. [file 40814_2023_1387_MOESM7_ESM.docx]

Additional file 7. Observation protocol to measure fidelity to approach

**RecoverED Process Evaluation**

**Observation Protocol for Audio Recordings**

Some intervention sessions will be audio-recorded as part of the embedded process evaluation. These audio recordings allow for re-examination of a particular intervention session in order to assess the rehabilitation support worker’s (RSW’s) adherence to the RecoverED treatment manual, specifically with regard to the ***intervention delivery approach***. Certain skills are essential to the quality of delivery and fall under 5 categories –

1. Person-centeredness
2. Personalisation and tailoring
3. Integration of activities to daily living
4. Involvement of family carers
5. Continuity of care

Please use the below observation protocol to rate the extent to which each skill was implemented in the particular session by the RSW delivering the session. Use the notes column at the end to document further feedback on the particular skill in question. Please do not note down any details that could personally identify either the participants or the RSW.

The following scale should be used to complete the rating –

**0 = Missed opportunity.** Use this rating if the skill was rarely or never demonstrated (skill demonstrated < 25% of the time)

**1 = Sometimes.** Use this rating if the skill is occasionally demonstrated (skill demonstrated 25-75% of the time)

**2 = Always/almost always** - Use this rating if skill is consistently demonstrated (skill demonstrated > 75% of the time)

**N/A=** Insert N/A if the mentioned skill is not applicable to the current session.

| **Category** | **Skill** | **Not Applicable** | **Always/Almost Always** | **Sometimes** | **Missed Opportunity** | **Notes** |
| --- | --- | --- | --- | --- | --- | --- |
| **Person-centeredness** | Actively engaged the participant in the planned session using appropriate encouragement, guidance, and validation of effort. |  |  |  |  |  |
|  | Communicated with the patient and carer participants in a respectful and non-judgmental manner. |  |  |  |  |  |
|  | Prioritised the choices and preferences of the patient participant in the delivery of the planned session. |  |  |  |  |  |
| **Personalisation and tailoring** | Prioritised the elements of care that are important to the patient participant. |  |  |  |  |  |
|  | Revisited the intervention goals with the patient (or carer) participant during the session to ensure that the planned session is aligned with the goals. |  |  |  |  |  |
|  | Consulted actively with the carer participant to ensure that the planned session matched the patient participant’s background preferences and abilities. |  |  |  |  |  |
| **Integrating activities to daily functioning** | Actively tried to maintain the patient participant’s engagement and participation during the session. |  |  |  |  |  |
|  | Implemented activities in the session that were part of the patient participant’s daily or routine functioning, as opposed to abstract tasks. |  |  |  |  |  |
|  | Listened to feedback from the patient or carer participants if the activity was not engaging/to their liking and made appropriate modifications to suit their preference. |  |  |  |  |  |
|  | Ensured that the activities were implemented in a way would not be perceived as redundant to the patient participant’s daily functioning goals. |  |  |  |  |  |
| **Involvement of family carers** | Actively engaged the carer participant in the delivery of the planned session. |  |  |  |  |  |
|  | Sought the input of the carer participant on the relevance of the planned session or with problem-solving (if required) during the session. |  |  |  |  |  |
|  | Ensured active listening to the carer participant’s concerns (if any), answered their questions to the best of their ability, and offered re-assurance. |  |  |  |  |  |
|  | Provided the carer participant with information, support, or resources when required. |  |  |  |  |  |
| **Continuity of care** | Made active efforts to build a trusting relationship with the patient participant by establishing rapport. |  |  |  |  |  |
|  | Actively listened to concerns (if any) expressed by the patient and/or carer participants during the session and responded appropriately, either through active listening, aiding with sense-making or offering re-assurances. |  |  |  |  |  |
|  | Used insights, gains and feedback from previous sessions during the current planned session to improve engagement and performance on the task/activity. |  |  |  |  |  |
